# Future health expenditure in the BRICS countries: a forecasting analysis for 2035

**DOI:** 10.1186/s12992-023-00947-4

**Published:** 2023-07-11

**Authors:** Pragyan Monalisa Sahoo, Himanshu Sekhar Rout, Mihajlo Jakovljevic

**Affiliations:** 1 Department of Analytical & Applied Economics, Utkal University, Bhubaneswar, India; 2 Department of Analytical and Applied Economics & Co-Coordinator, RUSA Centre of Excellence in Public Policy and Governance, Utkal University, Vani Vihar, Odisha 751 004 Bhubaneswar, India; 3 Institute of Advanced Manufacturing Technologies, Peter the Great St. Petersburg Polytechnic University, St. Petersburg, Russia; 4 Institute of Comparative Economic Studies, Hosei University Faculty of Economics, Tokyo, Japan; 5 Department of Global Health Economics and Policy, University of Kragujevac, Kragujevac, Serbia

**Keywords:** Health expenditure, Health spending, Emerging markets, BRICS, Forecasting, ETS

## Abstract

**Background:**

Accelerated globalization especially in the late 1980s has provided opportunities for economic progress in the world of emerging economies. The BRICS nations’ economies are distinguishable from other emerging economies due to their rate of expansion and sheer size. As a result of their economic prosperity, health spending in the BRICS countries has been increasing. However, health security is still a distant dream in these countries due to low public health spending, lack of pre-paid health coverage, and heavy out-of-pocket spending. There is a need for changing the health expenditure composition to address the challenge of regressive health spending and ensure equitable access to comprehensive healthcare services.

**Objective:**

Present study examined the health expenditure trend among the BRICS from 2000 to 2019 and made predictions with an emphasis on public, pre-paid, and out-of-pocket expenditures for 2035.

**Methods:**

Health expenditure data for 2000–2019 were taken from the OECD iLibrary database. The exponential smoothing model in R software (*ets ()*) was used for forecasting.

**Results:**

Except for India and Brazil, all of the BRICS countries show a long-term increase in per capita PPP health expenditure. Only India’s health expenditure is expected to decrease as a share of GDP after the completion of the SDG years. China accounts for the steepest rise in per capita expenditure until 2035, while Russia is expected to achieve the highest absolute values.

**Conclusion:**

The BRICS countries have the potential to be important leaders in a variety of social policies such as health. Each BRICS country has set a national pledge to the right to health and is working on health system reforms to achieve universal health coverage (UHC). The estimations of future health expenditures by these emerging market powers should help policymakers decide how to allocate resources to achieve this goal.

## Background

Accelerated globalization, especially in the late 1980s, has provided opportunities for economic progress in the world of emerging economies [1, 2]. Given the widely acknowledged relationship between economic prosperity and better health [3], many decision-makers see BRICS (Brazil, Russia, India, China, South Africa) as an instrument for altering global health due to their expanding economies. While BRICS has traditionally focused on economic cooperation, the COVID-19 pandemic has highlighted the importance of collaboration among these nations in the realm of public health. BRICS plays a significant role in the field of health due to its member countries’ large populations, economic growth, and regional influence. “Intra-BRICS health cooperation” expanded at the start of the previous decade, when health ministers of BRICS countries decided to meet yearly on the side-lines of the World Health Assembly in 2012. The primary objectives of these meetings were to strengthen domestic health systems and support south-south co-operations, global health partnerships, and international organizations like WHO and UNAIDS in realizing global goals such as the sustainable development goals (SDGs) [4]. The significance of BRICS in post-pandemic health lies in its potential to foster cooperation, leverage collective resources, and address common health challenges.

While implementation of the SDGs led to an increase in global health spending reaching $7.9 trillion in 2017 and is projected to reach $11 trillion by 2030 [5], the percentage share of BRICS nations in global health expenditure grew in comparison to many developed countries, which witnessed a decline [6]. Despite the many challenges faced by their health systems, the health expenditure has increased as a result of their economic prosperity and the various steps each country has adopted to improve its health systems [7–11]. Future projection of health spending also shows a general long-term trend towards expansion in both the total health spending as a percentage of GDP and per capita health spending in these emerging economies [12].

However, only rising health spending is not a representative indicator of the betterment of health indices [13, 14], which is the case with the BRICS. Their health spending growth is mostly driven by an ageing population and the double burden of diseases [15–17]. Despite the rise in total health spending, health security is still a distant dream in these countries due to low public health expenditure [18], the rising cost of healthcare [19], and heavy out-of-pocket expenditure [20]. In order to address the problem of regressive health expenditure, safeguard their citizens from intolerably high healthcare expenditure and ensure access to comprehensive, non-discriminatory healthcare services, there is a need for changing the composition and pattern of healthcare spending. Public funding of healthcare and effective risk-sharing in healthcare consumption while reducing the share of out-of-pocket expenditure is required to ensure equity in healthcare provision [14].

In the past, health was viewed as a luxury good, thus its provision was left to the market mechanism. However, it has been repeatedly demonstrated and is now generally acknowledged throughout the health economics literature that health is a necessity with an income elasticity of less than 1 [17, 21]. Therefore, the government should not leave healthcare entirely in the hands of private entities but should provide a subsidized and affordable form of healthcare service to the populace, because many people have become impoverished because of the snowballing rising cost of healthcare, which has resulted in many lives being lost in the long run [3]. Increased government participation in healthcare financing also has many positive health and health system consequences such as increased demand for public health services [22], fulfilment of the UHC target [23], improvement in the efficiency of public health programmes [24], and better health outcomes [25].

The difference between the BRICS and its developed counterparts is that whereas healthcare is largely publicly funded in the developed nations, it is not the case in any of the BRICS nations due to a lack of public investment. The public sector contribution to the total health spending in such middle- and low-income category countries is very low [5]. Among all the BRICS nations, only in Russia, the largest share of health spending is done by the state with a share of more than 80%, since the government is the major provider of medical care for the population in the Russian Federation and the care is provided on the basis of state guarantees of free medical care programmes [26]. Administrative structure and political dynamics have an impact on health spending, particularly in democratic republics such as India. The Indian case is particularly relevant because of its federal structure. Health is recognized as a state subject in India. In other words, health is essentially the responsibility of state governments. As a result, public health spending differs among states depending on the priorities and political will of the provincial governments [21].

Nevertheless, the expansion of coverage and the move towards UHC – after the introduction of SDG in 2015 – are shifting the ratio of public to private financing sources in these countries [5]. In India, public health expenditure in absolute terms has exhibited a consistent upward trend, indicating an overall increase since 1995. However, when examining health expenditure as a share of the country’s GDP, it has remained relatively stagnant. Other BRICS countries also increased their total health expenditure substantially. In Brazil, it grew by 3%, China – by 2%, South Africa – by 1.5%, and Russia – by 1.2% during the same time [10]. While increases in public health spending in some countries can be seen positively, they can also have a detrimental impact. The heavy reliance on public spending can be a cause of concern for financial sustainability [27]. The budgetary space available to governments in these developing countries is already insufficient to cover all the financial requirements for the universal health and well-being of their population [25]. Hence, financial resources for health must be prepaid and pooled across individuals via insurance coverage in order to ensure that health spending does not cause financial hardship for the national budget and impoverishment for the citizens [5]. Expanding risk pooling is one of the major international topics in the development of UHC [14, 28].

BRICS countries accept and endorse the conceptualization of UHC. However, analysis reveals that BRICS countries’ conceptualization of UHC seems to be driven by specific country circumstances and national needs. While some of them are focusing on increasing pooled resources, others are focusing on mainly the public provision of healthcare. China is enhancing UHC through the rapid injection of government finance into social health insurance to cover rural and urban residents [14]. Brazil built its own publicly funded healthcare system called Sistema Único de Saúde (SUS) in an effort to achieve universal healthcare coverage. However, the popularity of private health insurance is more prevalent than public health insurance in the country [29]. India is channelling the world’s largest health insurance programme named Ayushman Bharat under the Pradhan Mantri Jan Arogya Yojana (PM-JAY). In South Africa, the health sector is not well-funded and out-of-pocket spending is high. For UHC watchers, all eyes are on the government’s universal health financing system, the national health insurance programme, which is being rolled out over the next few years [4].

Health outcomes and access to care may be impacted by the availability of both public and pre-paid resources. Previous international research has demonstrated that when nations become wealthier, their citizens experience more financial hardship because they depend more on out-of-pocket spending [25]. The challenge for policymakers is in ensuring that additional healthcare resources are directed through the required pooled prepayment plans rather than out-of-pocket payments, which create access disparities across different socioeconomic strata [25].

The COVID-19 pandemic worsened the deficiencies of the healthcare system by causing a severe shortage of resources, infrastructure, and health workforce in these countries. More than 30% of COVID-19 infections and more than 20% of the pandemic deaths occurred in BRICS countries during the first wave of the virus [30]. Henceforth, nations are prioritizing healthcare with an aim to adopt effective capacity development methods to mitigate future health concerns. These measures will make the demand for adequate funding even more urgent [30–32].

All of these nations must therefore make it a top priority to explore potential possibilities for the growth of health spending and their implications for public finances. Stepping towards this direction, policymakers around the world are attempting to understand how health expenditure may change and to set a course for policy, whether it be due to concerns about the fiscal sustainability of public expenditure, rising health prices, the productivity of the health sector, financial strains on patients and families, or extending coverage. To promote the achievement of national and international health goals, comprehensive and comparable estimates of health spending in each nation are crucial inputs for health policy and planning. This requires understanding historical trends and predicting future trends in health spending [13, 33]. Effective health policy must consider anticipated future health expenditure and their source. Without careful planning and a lack of public funding for health can result in people having less access to care and relying more on out-of-pocket payments to purchase healthcare [34]. When looking at healthcare finance from the perspective of financing schemes, it is possible to conduct a more thorough analysis that also enables the monitoring of how financing patterns are changing over time [35], whether it is becoming progressive with a steady rise in pre-paid and pooled financing or regressive with an increase in out-of-pocket spending.

While there have been several studies projecting health expenditure, there is a noticeable lack of studies specifically focusing on the BRICS countries. Only one study conducted by Jakovljevic et al. [12] has projected healthcare spending among the BRICS nations, but it concentrated solely on estimating health expenditure until 2030. In contrast, the current study aims to project health expenditure until 2035. The study addresses the following research question;

### Research Question

A) What will be the long-term trend and pattern of future health expenditure for the BRICS bloc?

To answer the above research question, the primary objective of the study is to thoroughly examine long-term trends and make accurate predictions of health expenditure patterns among the BRICS up to 2035 with a special emphasis on government, pre-paid, and out-of-pocket expenditure. The timeframe for achieving SDG 2015’s goals 3.8 and 3.c i.e., UHC through effective financial risk protection and an increase in total healthcare spending is set to 2030. Forecasting healthcare expenditure patterns for 2035 will help to predict the target achievement or lack thereof among these nations. After the onset of COVID-19, it has become more crucial than ever before that healthcare is publicly financed, and risk pooling is especially important in low- and middle-income nations and newly emerging market economies such as BRICS. Expenditure forecasting will also serve in predicting how the countries will fare in this domain throughout the upcoming medium term.

## Methods

### Data

**Fig. 1:**
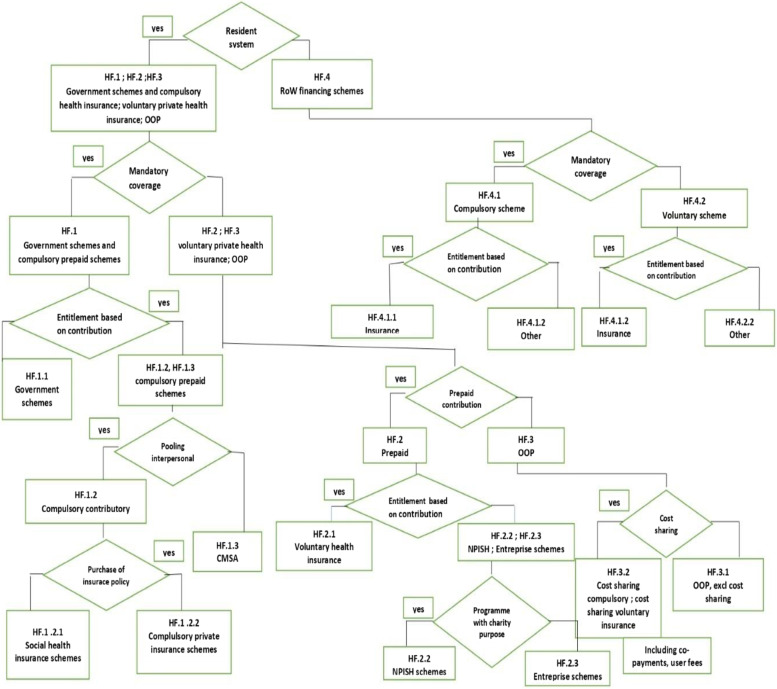
System of health accounts 2011 classification of health expenditure. Source: OECD/Eurostat/WHO [37]

The present study used the SHA 2011 classification of healthcare financing schemes, which is regarded as the international standard for the construction of National Health Accounts (NHA) and is also adopted by BRICS nations, to analyze and make projections on health expenditure. The SHA 2011 framework does away with the term “total health expenditure,“ but instead suggests using the term “current health expenditure”, which refers to the final consumption of healthcare goods and services by the government, non-profit organizations, and households, excluding the cost of fixed assets. Such an arrangement will enable the tracking of medical expenses precisely [8]. The SHA 2011 classification is given in Fig. 1. We retrieved health expenditure statistics from the OECD database [36] for the BRICS countries from 2000 to 2019. For the purpose of the analysis, all health expenditure is expressed in US dollars adjusted for inflation and purchasing power parity (PPP). As the database does not contain inflation and PPP-adjusted statistics for India and China, the current values were manually adjusted using PPP and CPI values from the OECD database for these countries.

### Methods

From 2000 to 2019, total health expenditure, government, pre-paid private, and out-of-pocket health expenditure were summarized using the mean, standard deviation, and percentage change. From 2020 to 2035, the paper estimated future total health expenditure, government, prepaid private, and out-of-pocket health expenditure in inflation-adjusted $PPP, per capita, as a percentage of GDP, and as a share of total current health expenditure. Time series analysis and exponential smoothing models [ETS] are employed in the forecasting processes.

The exponential smoothing approach was first developed by Robert Godwell Brown in 1956 and it was further expanded by Charles C. Holt in 1957. This method is widely used in the literature because it predicts by assigning a high weight to the nearest outcomes over time. The term “exponential smoothing” refers to the fact that the weights of the observations decrease exponentially as they get older. The ETS model takes into account the error, trend, and seasonal components of a given time series and evaluates 30 alternative models before selecting the best performing model to simulate the data. The error, trend, and seasonal components are the three major parameters, which can be additive (A), multiplicative (M), or none (N). The best performing model is determined by the lowest AIC, AICc, or BIC. The ETS technique is provided by the forecast package of R software outlined by Hyndman et al. [38] as an automatic forecasting model incorporating the foundations of exponential smoothing. These methods have a strong statistical foundation [39], are easy to apply, and have performed well in various forecasting cases [40]. Given the benefits of exponential smoothing methods and the large number of models available which can be chosen using information criteria (AIC, AICc, and BIC), the current study employs the best ETS model by using the *ets* function in R. The function by default uses the AICc to select an appropriate model. Unlike the *auto.arima* function, *ets* does not return forecasted values, it instead estimates model parameters and offers information about the fitted model. Hence, for forecasting the *forecast(ets ( ))* command was used.

## Results

Table 1 depicts the mean and standard deviation (in parentheses) of total health expenditure, health expenditure per capita and as a share of GDP, and the source of health expenditure per capita and share of health expenditure as a proportion of total for the years 2000 to 2019 for BRICS. All the absolute values are in inflation-adjusted $PPP. China accounted for the highest value of total health expenditure ($620762.90); however, it stands second lowest both in terms of health expenditure per capita ($449.47) and as a share of GDP (4.49%) among the BRICS group. The highest share of the total health expenditure is attributed to out-of-pocket expenses (46.77%), which is followed by health expenses by the government (44.71%), and the lowest share is of pre-paid private health expenditure (8.41%). The country with the highest health expenditure as a share of GDP is Brazil (8.46%), and the country has government health expenditure (42.87%) as a major share of the total health expenditure. The countries with the next highest health expenditure per capita are South Africa which spends 8.33% of its GDP on health and Russia spending 5.11% of its GDP on health. While Russia has government health expenditure (60.37%) as its major source, a majority of the health expenditure can be attributed to pre-paid private health expenditure (48.02%) in South Africa. India has the lowest value of health expenditure as a share of GDP (3.64%) and the major source is out-of-pocket expenditure (66.84%), which is higher than any other BRICS country.

**Table 1:** Descriptive statistics (Mean and Standard Deviation)

Country	Total health expenditure (HFTOT)			Source of health expenditure			Share of health expenditure		
	$PPP (in millions)	Per Capita (in $PPP millions)	% of GDP	Total government health expenditure (in $PPP millions)	Total pre-Paid private health expenditure (in $PPP millions)	Total out-of-pocket health expenditure (in $PPP millions)	Government health expenditure (% of CHE)	Pre-paid private health Expenditure (% of CHE)	Out-of-pocket health expenditure (% of CHE)
**Brazil**	324242.60 (53643.74)	1699.10 (377.72)	8.46 (0.58)	138969.50 (22522.92)	81571.9 (4891.61)	101925.3 (31052.65)	42.87 (1.24)	25.67 (3.52)	30.75 (4.63)
**Russia**	160686.50 (62441.05)	1202.835 (313.95)	5.11 (0.29)	97269.86 (38,014)	6619.312 (1441.79)	56744.82 (25767.13)	60.37 (2.07)	5.16 (3.04)	34.40 (3.18)
**India**	266332.50 (20893.18)	220.34 (30.05)	3.64 (0.43)	64991.27 (7593.27)	22262.13 (4218.01)	179018.50 (28509.41)	24.66 (4.41)	8.46 (2.06)	66.84 (6.33)
**China**	620762.90 (265168.2)	449.47 (178.48)	4.49 (0.44)	308405.60 (195201.7)	46818.87 (16499.71)	265118.40 (60959.18)	44.71 (13.68)	8.41 (3.22)	46.77 (10.88)
**South Africa**	51431.52 (12058.4)	990.73 (159.73)	8.33 (0.52)	22572.53 (7174.42)	24556.73 (5242.03)	4302.27 (433.21)	42.94 (4.40)	48.02 (1.69)	9.02 (3.02)

*PPP *Purchasing Power Parity, *CHE *Current Health Expenditure

**Fig. 2:**
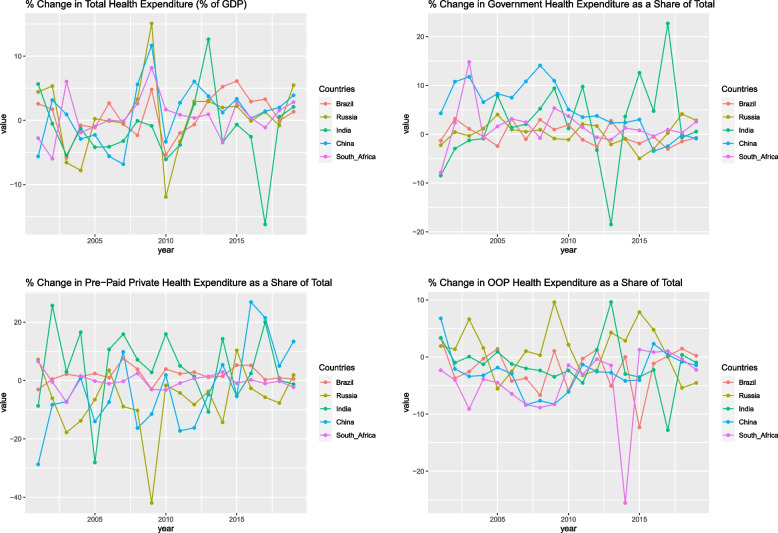
Change in total health expenditure as a share of GDP and share of health expenditure as a share of total (in %)

Figure 2 depicts the percentage change in total health expenditure as a share of GDP and health expenditure as a share of the total for the period 2000 to 2019.

As can be seen, there is no clear and consistent pattern in the BRICS bloc’s change in health expenditure and its source. India, Russia, and South Africa experienced the greatest fluctuation in the group. China and Brazil have a more stable trend.

**Fig. 3:**
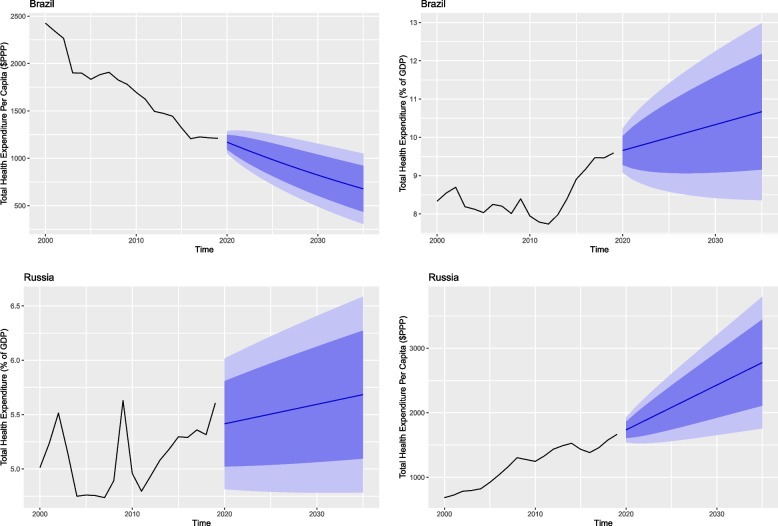
Total health expenditure per capita and percentage of GDP for Brazil and Russia

**Fig. 4:**
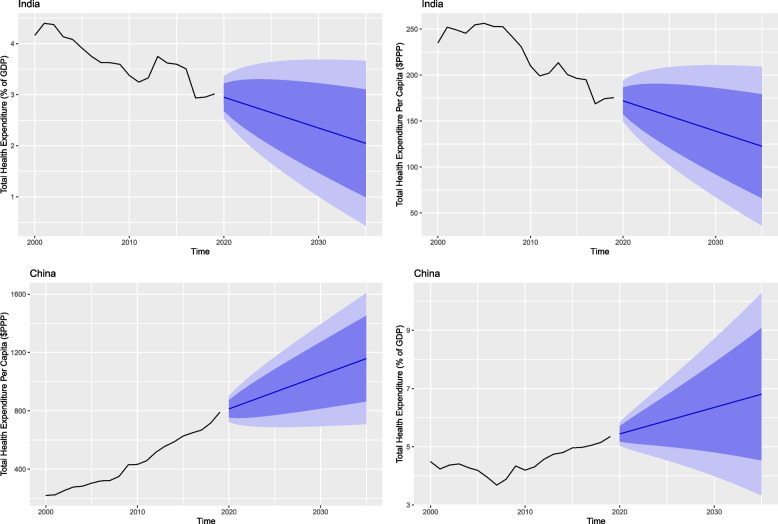
Total health expenditure per capita and percentage of GDP for India and China

**Fig. 5:**
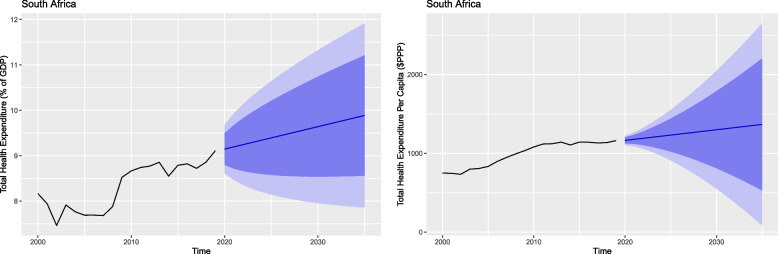
Total health expenditure per capita and percentage of GDP for South Africa

Figures 3, 4 and 5 show total health expenditure per capita and as a share of GDP for the BRICS countries. These figures depict the projected change in per capita health expenditure between 2020 and 2035.

Table 2 shows in 2035 all the member countries will witness a rise in their health expenses both in per capita and percentage of their GDP except for Brazil and India. Brazil which is the current highest spender in health – both per capita and share of GDP – is also expected to be the highest spender in health in terms of GDP share in 2035 with 10.67% (95% PI 8.36, 12.98). However, it is expected to witness a decrease in its per capita expenditure in 2035 with $677.82 (95% Prediction Interval [PI] 305.60, 1050.03) and is expected to be surpassed by Russia.

**Table 2:** Forecasted health expenditure for the years 2020, 2025, 2030 and 2035 (Per Capita and % of GDP)

Country	Health expenditure per capita				Health expenditure % of GDP			
	2020	2025	2030	2035	2020	2025	2030	2035
**Brazil**	1170.28(1052.04, 1288.52)	985.94(721.40, 1250.47)	822.58(490.73, 1154.43)	677.82(305.60, 1050.03)	9.66(9.08, 10.23)	9.99(8.58, 11.41)	10.33(8.41, 12.24)	10.67(8.36, 12.98)
**Russia**	1734.27(1542.09, 1926.45)	2081.95(1561.07, 2602.83)	2429.62(1654.61, 3204.64)	2777.30(1756.76, 3797.84)	5.41(4.81, 6.01)	5.50(4.79, 6.21)	5.59(4.78, 6.40)	5.68(4.78, 6.58)
**India**	172.00(149.72, 194.28)	155.54(102.40, 208.67)	139.08(67.29, 210.86)	122.61(36.10, 209.13)	2.94(2.53, 3.36)	2.64(1.65, 3.64)	2.34(1.00, 3.68)	2.04(0.43, 3.66)
**China**	813.51(722.32, 904.69)	928.55(688.21, 1168.89)	1043.59(694.66, 1392.53)	1158.64(708.95, 1608.33)	5.43(5.03, 5.84)	5.89(4.53, 7.25)	6.35(3.99, 8.71)	6.80(3.32, 10.28)
**South Africa**	1163.93(1105.48, 1222.38)	1231.50(910.36, 1552.63)	1299.06(551.15, 2046.98)	1366.63(84.05, 2649.20)	9.14(8.61, 9.68)	9.39(8.14, 10.64)	9.64(7.95, 11.32)	9.88(7.85, 11.91)

Russia is expected to spend $2777.30 (95% PI 1756.76, 3797.84) and become the highest per capita spender. India is the only country to witness a decline in both health expenditure per capita and as a share of its GDP in 2035 with a per capita value of $122.61 (95% PI 36.10, 209.13) and a share of GDP value of 2.04% (95% PI 0.43, 3.66). China is also expected to witness a rise in its per capita health expenditure with $1158.64 (95% PI 708.95, 1608.33) and GDP share with 6.80% (95% PI 3.32, 10.82). South Africa is expected to be the second highest spender in health per capita with a value of $1366.63 (95% PI 84.05, 2649.29), as well as a share of its GDP with a value of 9.88% (95% PI 7.85, 11.91) (Table 2).

Tables 3 and 4 show the sources of health expenditure per capita and their respective shares of health expenditure for the years 2020, 2025, 2030, and 2035. The highest government health expenditure per capita in 2020 is observed for Russia, at $1064.65 (95% PI 922.69, 1221.89), followed by South Africa at $578.07 (95% PI 509.01, 655.13), China at $477.82 (95% PI 410.79, 554.46), Brazil at $ 473.20 (95% PI 421.83, 530.09) and lastly India at $58.38 (95% PI 51.72, 65.89). In 2035, it is expected that the highest government health expenditure per capita will be observed for Russia, at $1936.32 (95% PI 1164.82, 2840.36), followed by China at $1327.36 (95% PI 280.46, 3436.56), South Africa at $939.39 (95% PI 588.08, 1432.44), Brazil at $253.08 (95% PI 165.29, 383.79) and India at $62.84 (95% PI 34.46, 114.06) (Table 3). In terms of the share of government health expenditure, the highest value in 2020 is observed for Russia, with 61.25% (95% PI 58.25%, 64.16%), followed by China with 55.64% (95% PI 52.67%, 58.56%), South Africa with 48.08% (95% PI 45.18%, 51%), Brazil with 40.24% (95% PI 38.47%, 42.04%), and lastly India with 33.76% (95% PI 28.63%, 39.31%). Russia, India, and South Africa are expected to experience a small and steady rise in government share in health expenditure in 2035, except Brazil and China, which will undergo a decline in government expenditure. In 2035, the share of government health expenditure for Russia is projected to be 62.71% (95% PI 50.52%, 73.47%), followed by South Africa with 49.50% (95% PI 45.95%, 53.05%), China with 48.55% (95% PI 1.59%, 98.22%), India with 44.17% (95% PI 22.14%, 68.75%), and Brazil with 31.91% (95% PI 18.26%, 49.58%) (Table 4).

**Table 3:** Source of health expenditure per capita for the years 2020, 2025, 2030, and 2035

Country	Government health expenditure per capita				Pre-paid private health expenditure per capita				Out-of-pocket health expenditure per capita			
	2020	2025	2030	2035	2020	2025	2030	2035	2020	2025	2030	2035
**Brazil**	473.20(421.83, 530.09)	385.40(297.90, 495.90)	312.77(220.32, 440.45)	253.08(165.29, 383.79)	369.38(333.5017 408.78)	333.76(259.23, 427.79)	301.35(213.41, 422.35)	271.91(178.81, 409.36)	260.67(224.67, 302.08)	186.65(155.62, 223.57)	133.05(107.80, 164.01)	94.54(74.68, 119.57)
**Russia**	1064.65(922.69, 1221.89)	1320.74(938.05, 1790.73)	1613.19(1034.18, 2326.22)	1936.32(1164.82, 2840.36)	36.68(26.67, 50.41)	31.32(14.31, 68.28)	26.74(9.23, 76.94)	22.82(6.31, 81.85)	626.44(588.85, 666.06)	705.31(604.5, 5819.74)	769.53(622.85, 943.31)	820.56(633.99, 1048.84)
**India**	58.38(51.72, 65.89)	59.83(43.44, 82.31)	61.32(38.49, 97.41)	62.84(34.46, 114.06)	19.78(16.82, 23.25)	19.85(16.88, 23.34)	19.87(16.89, 23.38)	19.88(16.89, 23.40)	93.92(77.64, 113.54)	83.71(52.42, 133.19)	74.59(39.51, 139.96)	66.46(30.83, 142.08)
**China**	477.82(410.79, 554.46)	686.68(389.91, 1152.84)	967.32(345.00, 2185.23)	1327.36(280.46, 3436.56)	82.35(63.50, 106.68)	212.83(58.43, 716.09)	527.84(32.15, 3413.73)	1192.90(12.20, 4878.37)	288.64(260.25, 319.93)	348.71(273.87, 442.09)	420.17(304.33, 574.70)	504.68(343.91, 728.85)
**South Africa**	578.07(509.01, 655.13)	682.67(506.34, 908.01)	802.77(539.84, 1160.43)	939.39(588.08, 1432.44)	536.47(508.33, 565.97)	544.29(432.61, 680.49)	552.20(351.96, 845.63)	560.21(273.63, 1078.45)	62.30(54.17, 71.62)	54.10(47.03, 62.21)	46.97(40.83, 54.02)	40.77(35.43, 46.90)

**Table 4:** Share of health expenditure for the years 2020, 2025, 2030, and 2035 (% of CHE)

Country	Government health expenditure (% of CHE)				Pre-paid private health expenditure (% of CHE)				Out-of-pocket health expenditure (% of CHE)			
	2020	2025	2030	2035	2020	2025	2030	2035	2020	2025	2030	2035
**Brazil**	40.24(38.47, 42.04)	37.37(32.05, 43.02)	34.59(24.96, 45.68)	31.91(18.26, 49.58)	32.11(30.55, 33.72)	35.37(33.19, 37.61)	38.77(36.05, 41.56)	42.28(39.07, 45.54)	23.05(21.30, 24.91)	19.96(18.38, 21.64)	17.18(15.78, 18.69)	14.73(13.48, 16.06)
**Russia**	61.25(58.25, 64.16)	61.73(54.32, 68.64)	62.22(52.14, 71.35)	62.71(50.52, 73.47)	2.08(1.56, 2.78)	1.37(0.69, 2.72)	0.90(0.35, 2.29)	0.59(0.19, 1.83)	39.37(35.77, 43.09)	41.88(38.19, 45.67)	44.44(40.67, 48.28)	47.03(43.18, 50.91)
**India**	33.76(28.63, 39.31)	37.11(24.47, 51.81)	40.59(22.96, 61.04)	44.17(22.14, 68.75)	12.32(10.20, 14.81)	14.81(12.32, 17.70)	17.70(14.81, 21.01)	21.01(17.69, 24.75)	53.57(47.43, 59.61)	47.52(33.08, 62.38)	41.53(23.79, 61.78)	35.79(17.08, 60.13)
**China**	55.64(52.67, 58.56)	53.28(29.07, 76.05)	50.92(8.78, 91.80)	48.55(1.59, 98.22)	9.36(7.22, 12.04)	11.59(3.82, 30.23)	12.42(1.78, 52.60)	12.71(0.90, 69.82)	34.00(30.41, 37.77)	28.19(20.79, 37.00)	23.03(14.78, 34.05)	18.57(10.57, 30.57)
**South Africa**	48.08(45.18, 51.00)	48.83(45.85, 51.83)	49.26(46.04, 52.48)	49.50(45.95, 53.05)	46.24(43.50, 49.01)	45.26(42.18, 48.37)	44.28(40.90, 47.70)	43.30(39.66, 47.01)	5.12(4.48, 5.85)	4.25(3.71, 4.86)	3.63(3.17, 4.15)	3.16(2.76, 3.62)

*CHE *Current Health Expenditure; SHA 2011 considers CHE as Total Health Expenditure

In terms of pre-paid private expenditure per capita, the highest expenditure in 2020 is expected to be observed by South Africa at $536.47 (95% PI 508.33, 565.97), followed by Brazil, at $369.38 (95% PI 333.5017 408.78), China at $82.35 (95% PI 63.50, 106.68), Russia at $36.68 (95% PI 26.67, 50.41), and India at $19.78 (95% PI 16.82, 23.25). In 2035, the pre-paid private expenditure per capita in China is expected to increase and be the highest, at $1192.90 (95% PI 12.20, 4878.37), followed by South Africa at $560.21 (95% PI 273.63, 1078.45), Brazil at $271.91 (95% PI 178.81, 409.36), Russia at $22.82 (95% PI 6.31, 81.85), and India at $19.88 (95% PI 16.89, 23.40) (Table 3).

In terms of the share of pre-paid private health expenditure, the highest in 2020 is expected to be observed by South Africa, with 46.24% (95% PI 43.50%, 49.01%), followed by Brazil with 32.11% (95% PI 30.55%, 33.72%), India with 12.32% (95% PI 10.20%, 14.81%), China with 9.36% (95% PI 7.22%, 12.04%) and Russia with 2.08% (95% PI 1.56%, 2.78%). In 2035, South Africa and Brazil are expected to have the highest share of pre-paid private expenditure, with 43.30% (95% PI 39.66%, 47.01%) for South Africa and 42.28% (95% PI 39.07%, 45.54%) for Brazil. China and India are expected to have a much smaller share of pre-paid private expenditure albeit the increase in expenditure, 21.01% (95% PI 17.69%, 24.75%) for India and 12.71% (95% PI 0.90%, 69.82%) for China, while Russia is expected to have the lowest share of pre-paid private expenditure, at 0.59% (95% PI 0.19%, 1.83%) (Table 4).

The highest out-of-pocket expenditure per capita in 2020 is expected to be observed by Russia, at $626.44 (95% PI 588.85, 666.06), followed by Brazil at $260.67 (95% PI 224.67, 302.08), China at $288.64 (95% PI 260.25, 319.93), India at $93.92 (95% PI 77.64, 113.54) and lastly South Africa at $62.30 (95% PI 54.17, 71.62). In 2035, the out-of-pocket expenditure per capita in Russia is expected to increase and to again be the highest at $820.56 (95% PI 633.99, 1048.84), followed by China at $504.68 (95% PI 343.91, 728.85), Brazil at $94.54 (95% PI 74.68, 119.57), India at $66.46 (95% PI 30.83, 142.08) and South Africa at $40.77 (95% PI 35.43, 46.90) (Table 3). In terms of the out-of-pocket share of health expenditure, the highest in 2020 is expected to be observed by India at 53.57% (95% PI 47.43%, 59.61%), followed by Russia at 39.37% (95% PI 35.77%, 43.09%), China at 34% (95% PI 30.41%, 37.77%), Brazil at 23.05% (95% PI 21.30%, 24.91%) and South Africa at 5.12% (95% PI 4.48%, 5.85%). In 2035, India and Russia are expected to have the highest out-of-pocket expenditure share, 47.03% (95% PI 43.18%, 50.91%) for Russia and 35.79% (95% PI 17.08, 60.13) for India, followed by China with 18.57% (95% PI 10.57%, 30.57%), Brazil with 14.73% (95% PI 13.48%, 16.06%) and South Africa with 3.16% (95% PI 2.76%, 3.62%) (Tables 4, 5, 6, 7, 8 and 9).

**Table 5:** ETS model information and parameter estimates with their error measures for Brazil

Indicator	Method	ME	RMSE	MAE	MPE	MAPE	MASE	ACF1
**Total Health Expenditure (% of GDP)**	ETS(A,A,N) α = 0.99; β = 0.00	-0.00	0.26	0.21	-0.11	2.58	0.93	0.18
**Total Health Expenditure Per Capita ($PPP)**	ETS(M,Ad,N) α = 0.98; β = 0.00	-12.06	83.95	54.32	-0.68	3.33	0.73	-0.03
**Government Health Expenditure Per Capita ($PPP)**	ETS(A,A,N) α = 0.87; β = 0.00	0.00	0.05	0.04	-0.11	2.50	0.84	0.00
**Pre-Paid Private Health Expenditure Per Capita ($PPP)**	ETS(A,A,N) α = 0.99; β = 0.00	0.00	0.05	0.03	-0.09	1.46	0.93	0.12
**Out-of-Pocket Health Expenditure Per Capita ($PPP)**	ETS(A,A,N) α = 0.30; β = 0.00	0.00	0.07	0.05	-0.14	2.40	0.79	0.29
**Government Health Expenditure (% of CHE)**	ETS(A,A,N) α = 0.66; β = 0.21	-0.01	0.03	0.02	2.26	10.49	0.94	-0.03
**Pre-Paid Private Health Expenditure (% of CHE)**	ETS(A,A,N) α = 0.39; β = 0.00	0.00	0.03	0.02	-0.19	2.60	0.82	0.22
**Out-of-Pocket Health Expenditure (% of CHE)**	ETS(A,A,N) α = 0.00; β = 0.00	-0.00	0.04	0.03	-0.07	4.83	0.89	0.21

*ETS* Exponential smoothing model; error, trend, and seasonal components are the three major parameters, which can be additive (A), multiplicative (M), or none (N); α = smoothing parameter for the level component; β = smoothing parameter for trend component; *ME *Mean Error, *RMSE *Root Mean Squared Error, *MAE *Mean Absolute Error, *MPE *Mean Percentage Error, *MAPE *Mean Absolute Percentage Error, *MASE *Mean Absolute Scaled Error, *ACFI *Auto-correlation of Errors at lag 1

**Table 6:** ETS model information and parameter estimates with their error measures for Russia

Indicator	Method	ME	RMSE	MAE	MPE	MAPE	MASE	ACF1
**Total Health Expenditure (% of GDP)**	ETS(A,A,N) α = 0.29;β = 0.00	0.00	0.27	0.22	-0.28	4.24	1.05	0.29
**Total Health Expenditure Per Capita ($PPP)**	ETS(M,A,N) α = 0.99;β = 0.00	-15.21	65.11	51.61	-1.26	4.42	0.71	0.37
**Government Health Expenditure Per Capita ($PPP)**	ETS(A,A,N) α = 0.99;β = 0.00	0.00	0.08	0.07	0.11	3.93	0.77	0.43
**Pre-Paid Private Health Expenditure Per Capita ($PPP)**	ETS(A,A,N) α = 0.99;β = 0.00	0.00	0.15	0.09	-0.02	2.01	1.06	0.02
**Out-of-Pocket Health Expenditure Per Capita ($PPP)**	ETS(A,Ad,N) α = 0.99;β = 0.01	0.00	0.03	0.03	0.02	1.06	0.41	0.39
**Government Health Expenditure (% of CHE)**	ETS(A,A,N) α = 0.99;β = 0.00	0.00	0.06	0.05	-1.85	11.47	1.00	0.42
**Pre-Paid Private Health Expenditure (% of CHE)**	ETS(A,A,N) α = 0.95;β = 0.00	0.00	0.13	0.08	-0.04	2.75	0.76	0.01
**Out-of-Pocket Health Expenditure (% of CHE)**	ETS(A,A,N) α = 0.02;β = 0.00	0.00	0.07	0.06	-1.89	10.24	1.08	0.53

*ETS* Exponential smoothing model; error, trend, and seasonal components are the three major parameters, which can be additive (A), multiplicative (M), or none (N); α = smoothing parameter for the level component; β = smoothing parameter for trend component; *ME *Mean Error, *RMSE *Root Mean Squared Error, *MAE *Mean Absolute Error, *MPE *Mean Percentage Error, *MAPE *Mean Absolute Percentage Error, *MASE *Mean Absolute Scaled Error, *ACFI *Auto-correlation of Errors at lag 1

**Table 7:** ETS model information and parameter estimates with their error measures for India

Indicator	Method	ME	RMSE	MAE	MPE	MAPE	MASE	ACF1
**Total Health Expenditure (% of GDP)**	ETS(A,A,N) α = 0.96;β = 0.00	0.00	0.19	0.13	-0.21	3.66	0.88	0.06
**Total Health Expenditure Per Capita ($PPP)**	ETS(A,A,N) α = 0.96;β = 0.00	0.16	10.17	7.64	-0.15	3.65	0.92	0.07
**Government Health Expenditure Per Capita ($PPP)**	ETS(A,A,N) α = 0.96;β = 0.03	0.00	0.06	0.04	-0.04	0.91	0.95	0.13
**Pre-Paid Private Health Expenditure Per Capita ($PPP)**	ETS(A,Ad,N) α = 0.00;β = 0.00	0.00	0.07	0.05	0.03	0.88	0.66	-0.12
**Out-of-Pocket Health Expenditure Per Capita ($PPP)**	ETS(A,A,N) α = 0.99;β = 0.00	-0.01	0.09	0.06	0.11	1.69	0.88	0.01
**Government Health Expenditure (% of CHE)**	ETS(A,A,N) α = 0.99;β = 0.00	0.00	0.11	0.08	-1.04	7.35	0.93	0.11
**Pre-Paid Private Health Expenditure (% of CHE)**	ETS(A,A,N) α = 0.00;β = 0.00	0.00	0.10	0.08	-0.12	3.23	0.72	0.08
**Out-of-Pocket Health Expenditure (% of CHE)**	ETS(A,A,N) α = 0.99;β = 0.00	0.01	0.11	0.07	-3.21	16.15	0.82	0.03

*ETS* Exponential smoothing model; error, trend, and seasonal components are the three major parameters, which can be additive (A), multiplicative (M), or none (N); α = smoothing parameter for the level component; β = smoothing parameter for trend component; *ME *Mean Error, *RMSE *Root Mean Squared Error, *MAE *Mean Absolute Error, *MPE*: Mean Percentage Error, *MAPE *Mean Absolute Percentage Error, *MASE *Mean Absolute Scaled Error, *ACFI *Auto-correlation of Errors at lag 1

**Table 8:** ETS model information and parameter estimates with their error measures for China

Indicator	Method	ME	RMSE	MAE	MPE	MAPE	MASE	ACF1
**Total Health Expenditure (% of GDP)**	ETS(A,A,N) α = 0.99;β = 0.13	0.04	0.19	0.14	0.75	3.32	0.87	0.07
**Total Health Expenditure Per Capita ($PPP)**	ETS(M,A,N) α = 0.99;β = 0.00	6.68	22.26	15.85	0.64	3.57	0.53	0.07
**Government Health Expenditure Per Capita ($PPP)**	ETS(A,A,N) α = 0.99;β = 0.20	-0.01	0.08	0.06	0.60	1.93	0.50	0.05
**Pre-Paid Private Health Expenditure Per Capita ($PPP)**	ETS(A,A,N) α = 0.77;β = 0.44	0.04	0.12	0.10	-0.75	2.04	0.97	-0.07
**Out-of-Pocket Health Expenditure Per Capita ($PPP)**	ETS(A,A,N) α = 0.95;β = 0.00	0.00	0.05	0.04	-0.02	1.18	0.68	0.01
**Government Health Expenditure (% of CHE)**	ETS(A,A,N) α = 0.99;β = 0.86	0.00	0.05	0.04	-7.54	19.99	0.39	0.03
**Pre-Paid Private Health Expenditure (% of CHE)**	ETS(A,Ad,N) α = 0.70;β = 0.58	0.02	0.12	0.11	-0.93	4.62	0.85	-0.01
**Out-of-Pocket Health Expenditure (% of CHE)**	ETS(A,A,N) α = 0.99;β = 0.00	0.00	0.07	0.05	-6.95	26.71	0.67	0.34

*ETS* Exponential smoothing model; error, trend, and seasonal components are the three major parameters, which can be additive (A), multiplicative (M), or none (N); α = smoothing parameter for the level component; β = smoothing parameter for trend component; *ME *Mean Error, *RMSE *Root Mean Squared Error, *MAE *Mean Absolute Error, *MPE *Mean Percentage Error, *MAPE *Mean Absolute Percentage Error, *MASE *Mean Absolute Scaled Error, *ACFI *Auto-correlation of Errors at lag 1

**Table 9:** ETS model information and parameter estimates with their error measures for South Africa

Indicator	Method	ME	RMSE	MAE	MPE	MAPE	MASE	ACF1
**Total Health Expenditure (% of GDP)**	ETS(A,A,N) α = 0.94;β = 0.00	0.00	0.24	0.18	-0.07	2.17	0.93	0.00
**Total Health Expenditure Per Capita ($PPP)**	ETS(A,A,N) α = 0.58;β = 0.56	-1.45	26.67	20.66	-0.14	2.20	0.73	0.03
**Government Health Expenditure Per Capita ($PPP)**	ETS(A,A,N) α = 0.96;β = 0.00	0.00	0.07	0.05	-0.04	1.95	0.89	0.11
**Pre-Paid Private Health Expenditure Per Capita ($PPP)**	ETS(A,A,N) α = 0.99;β = 0.26	0.00	0.03	0.02	0.23	0.98	0.82	0.03
**Out-of-Pocket Health Expenditure Per Capita ($PPP)**	ETS(A,A,N) α = 0.00;β = 0.00	0.00	0.06	0.05	-0.03	1.11	1.23	0.32
**Government Health Expenditure (% of CHE)**	ETS(A,Ad,N) α = 0.03;β = 0.03	0.01	0.05	0.04	-4.16	15.39	0.84	0.05
**Pre-Paid Private Health Expenditure (% of CHE)**	ETS(A,A,N) α = 0.23;β = 0.00	0.00	0.05	0.04	18.14	56.39	1.16	0.27
**Out-of-Pocket Health Expenditure (% of CHE)**	ETS(A,Ad,N) α = 0.00;β = 0.00	0.00	0.06	0.05	-0.18	1.98	0.84	0.32

*ETS* Exponential smoothing model; error, trend, and seasonal components are the three major parameters, which can be additive (A), multiplicative (M), or none (N); α = smoothing parameter for the level component; β = smoothing parameter for trend component; *ME *Mean Error, *RMSE *Root Mean Squared Error, *MAE *Mean Absolute Error, *MPE *Mean Percentage Error, *MAPE *Mean Absolute Percentage Error, *MASE *Mean Absolute Scaled Error, *ACFI *Auto-correlation of Errors at lag 1

## Discussion

BRICS countries have undertaken – or committed to – significant health-system reforms aimed at improving equity in health service use, quality, and financial protection, with the ultimate aim of achieving UHC. These health reforms are an important step towards translating the growing wealth of the BRICS into better health. These countries have taken different paths to the UHC, and they began moving along those paths at different times. Brazil and Russia began this process more than three decades ago. China and India are new players in the field, and South Africa has only recently begun its reform process [41]. Given the pressing issue of insufficient public health expenditure and the political nature of health reform, the BRICS bloc pledged in 2011 at their annual health ministers’ meeting to build their health financing systems as the foundation for UHC, following in the footsteps of the other WHO member countries [42].

The amount and source of health expenditure per capita in these countries appeared to significantly correlate with the health system outcomes [23]. Believing in the significance of health expenditure, the current paper attempted to uncover differences in health expenditure performance among BRICS countries, as well as to determine how expenditure in comparison to each other will change over time. The measure values themselves do not provide any information. They can only be interpreted in a comparative manner within the investigated group. A few interesting facts are revealed in the paper. First, Brazil, China, and Russia had comparatively better current and future health expenditure scenarios based on measured and predicted values. While India and South Africa both have significantly worse results than countries qualified for the first subgroup. The second intriguing finding is regarding the measured health expenditure value changes over time. For the period from 2000 to 2019, the percentage change in total health expenditure as a share of GDP and share of total health expenditure revealed no clear and consistent pattern. India, Russia, and South Africa saw the most variation in the group. China and Brazil are experiencing a more stable trend. However, out-of-pocket expenditure on health care has been declining in each country for the last two to three years, except for Russia, which has seen a slight increase. Except for India and Brazil, all of the BRICS countries show a long-term increase in health expenditure. Only India’s health expenditure is expected to decrease as a share of GDP in 2035. China appears to witness the steepest increase in per capita health expenditure until 2035, while Russia is expected to achieve the highest absolute values. A predominance of public funding is a necessary condition for achieving UHC. Health reforms have given the governments of the BRICS countries significant roles in the health sector, particularly in healthcare financing. However, private financing of health care continues to account for a sizable portion of total health expenditure. The 19-year average private financing, both pre-paid private and out-of-pocket, accounts for approximately 57%, 40%, 75%, 55%, and 57% of total health expenditure in Brazil, Russia, India, China, and South Africa, respectively. Russia stands out as the country with the greatest proportion of government spending in relation to its total health expenditure. Specifically, Russia allocates 60.37% of its total health spending through government channels. This observation aligns with the findings of Nikolina and Ratkin [26], who also reported Russia to have the highest government health spending relative to total health expenditure. However, it is worth noting that the present study diverges from Nikolina and Ratkin [26] in terms of the actual numerical estimates. While their research indicated government health spending to exceed 83%, the current study reveals a comparatively lower figure.

In certain instances, there are concerning trends in estimated health spending that could have significant implications for various countries. For example, India is expected to experience a decrease in total health spending as a percentage of its GDP. Given India’s large population, widespread poverty, and high disease burden, this reduction in health spending would result in less investment in human capital, leading to both health and economic consequences. Similarly, Russia is projected to witness an increase in out-of-pocket expenditure, coupled with a decline in the share of pre-paid coverage in total health expenditure. This shift in the health financing system could have regressive effects, potentially leading to a significant number of people being pushed into poverty due to healthcare expenses. South Africa, which has been actively working towards implementing a National Health Insurance system, is facing a potential setback with the expected decline in the share of pre-paid coverage. This reversal of progress could undermine the efforts made towards achieving universal health coverage. Moreover, both China and Brazil are anticipated to experience a decrease in the government’s share of total health expenditure. This reduction, unless compensated by a rise in pre-paid coverage, may result in health-related impoverishment for individuals in these countries. These trends carry inherent risks for epidemiological outcomes and the performance of health systems in these nations.

It is crucial for policymakers and stakeholders to address these challenges and ensure adequate investment in healthcare to safeguard the well-being of their populations and mitigate potential adverse effects on both health and economies. The noticeable positive transition in the health expenditure landscape among BRICS and globally can be attributed primarily to China. China’s population will age rapidly in the first half of the twenty-first century, making it the world’s fastest ageing large nation. Similar population trends have existed in Brazil since the 1980s, and in Russia for many decades. Unlike the others, India, as a still young large country, is likely to benefit from a demographic dividend from the expected 150 million labour force expansion [43, 44]. However, expenditure statistics do not provide much information about the cost-effectiveness and efficiency of the allocation of resources within these national health systems. As a result, it is reasonable to expect a wide range of individual success rates in these countries’ expansion of hospital networks, investments in medical education, and development of professional human capacities. Some of the BRICS’ inter-country differences are reflected in health inequities [45]. A typical example is that rural populations have far less affordable medical care than urban populations [6].

Despite the fact that the BRICS bloc was formed based on the economic size and potential of the underlying countries, there is no simple correlation between wealth and health in these countries. Some discovered a lack of responsiveness of health and health expenditure to economic prosperity [21], while others discovered an adverse effect of macroeconomic prosperity on health expenditure and population health in an environment of badly planned health policy [46]. Rapid economic growth has also caused major issues such as rapid urbanization has resulted in high levels of urban poverty [47]. Even though millions of BRICS residents have been lifted out of poverty over the last decade, the majority of the world’s poor still reside in BRICS or other emerging economies. If the general health of those living in the BRICS countries is to improve, political will and solutions, structural reforms, and massive financial investments will be required at both the national and global levels.

Although national governments in these countries have played an important role in experimenting with healthcare reforms, private financing continues to account for a significant portion of BRICS healthcare expenditure. China and India rely heavily on out-of-pocket expenses, while Brazil and South Africa rely heavily on private insurance. Brazilian health reforms were the result of a political movement to make health a constitutional right, whereas in the remaining four countries, health reforms were an attempt to improve the performance of the public system and reduce access inequities. The transition to UHC has been slow in these countries. The reforms in China and India have not adequately addressed the issue of heavy out-of-pocket payments. Negotiations between national and subnational entities have frequently been difficult. On one hand, through a constitutional delineation of responsibility, Brazil has been able to achieve good coordination between federal and state entities. On the other hand, poor coordination has always been an issue in Russia when it comes to the efficient use of resources [41].

It remains possible that the BRICS’ national health agendas will influence and direct the global health agenda. By 2035, China and India will have 35% of the world’s population [48], and with India already surpassing China as the most populous country, the scenario has become more likely [49]. WHO has acknowledged how BRICS have contributed to global health improvement through various domestic actions and policies on UHC and other health issues. Brazil has long been a leader in expanding healthcare access. Russia has made a significant commitment to combat non-communicable diseases. In 2013, the Chinese government set a goal of increasing the gross value of its healthcare sector to 1.31 trillion US dollars (US$). The South African government announced significant increase to its health budget in 2014. Other social determinants of health are also being addressed by the BRICS. The Brazilian government, for example, launched the Brasil sem Miséria (Brazil without Poverty) plan in 2011. India launched the world’s largest food subsidy programme in 2013. Such massive investments will certainly have an effect on the global health agenda if the resources are used efficiently to enhance effectiveness and achieve maximum health system outcomes [48, 50–52].

## Conclusion

There are a few distinct pathways of health expenditure among the five BRICS countries. China, as the primary driver of global economic growth, will be able to significantly increase its investment in the health sector across a range of indicators. Its chances of achieving tailored SDG targets in terms of universal health coverage are probably the highest of the group. Except for India and Brazil, all of the BRICS countries show a long-term increase in per capita PPP health expenditure. Only India’s health expenditure is expected to decrease as a share of GDP after the completion of the SDG years. China accounts for the steepest rise in per capita expenditure until 2035, while Russia is expected to achieve the highest absolute values. Each BRICS country has set a national pledge to the right to health and is working on health system reforms to achieve UHC. All, however, have a long way to go. The BRICS countries must succeed in moving towards UHC not only because they account for nearly half of the world population, but also because they can serve as role models for many other countries in their respective regions.

These estimations of future health expenditure by these emerging market powers should help policymakers decide how to allocate resources to achieve the UHC goal in the long-term. The findings of this current study may also serve as a catalyst for informed decision-making regarding healthcare expenditures within BRICS and other emerging market economies. It should also serve as a foundation for future health economics research on BRICS health sectors. While considerable efforts were made to uphold the quality and rigor of the estimates, it is important to acknowledge the limitations of the current study. One notable limitation is that the data used in this study only extends up to 2019, as data beyond that period was unavailable from the OECD iLibrary. Consequently, the study may not capture recent developments and emerging trends in the subject matter. Additionally, an important factor not accounted for in this study is the impact of the Covid-19 pandemic on health spending. Given the unprecedented nature of the pandemic and its potential to significantly influence healthcare systems and expenditures, the estimates presented in this study may be subject to inflation or distortion. It is crucial to recognize these limitations as they contribute to the contextual understanding of the findings and underscore the need for further research to encompass more recent data and incorporate the effects of Covid-19 on health spending.

## Data Availability

The datasets analyzed during the current study are available in the OECD iLibrary repository, 10.1787/health-data-en.

## Abbreviations

BRICS: Brazil, Russia, India, China, and South Africa
SDG: Sustainable Development Goal
UNAIDS: United Nations Programme on HIV/AIDS
SHA: System of Health Accounts
NHA: National Health Accounts
GDP: Gross Domestic Product
UHC: Universal Health Coverage
OECD: Organization for Economic Cooperation and Development
PM-JAY: Pradhan Mantri Jan Arogya Yojana
SUS: Sistema Único de Saúde
ETS: Exponential Smoothing Models
AIC: Akaike Information Criteria
AICc: Akaike’s Information Corrected Criterion
BIC: Bayesian Information Criterion

## References

[1] Jakovljevic M (2015). BRIC’s growing share of global health spending and their diverging pathways. Front Public Health..

[2] Jakovljevic M, Timofeyev Y, Ekkert N, Fedorova J, Skvirskaya G, Bolevich S (2019). The impact of health expenditures on public health in BRICS nations. J Sport Health Sci.

[3] Wahab A, Kefeli Z (2016). Projecting a long term expenditure growth in Healthcare Service: A literature review. Procedia Econ Financ.

[4] Harmer A (2014). The BRICS countries: a new force in global health?. Bull World Health Organ.

[5] Micah AE, Su Y, Bachmeier D, Chapin A, Cogswell IE, Crosby SW (2020). Health sector spending and spending on HIV/AIDS, tuberculosis, and malaria, and development assistance for health: progress towards sustainable development goal 3. Lancet.

[6] Jakovljevic MM (2016). Comparison of historical medical spending patterns among the BRICS and G7. JMed Econ.

[7] Reshetnikov V, Arsentyev E, Bolevich S, Timofeyev Y, Jakovljević M (2019). Analysis of the financing of Russian Health Care over the past 100 years. Int J Environ Res Public Health.

[8] Zhang S, Zhan H, Zhou L, Wang X (2019). Research on current curative expenditure among Lung Cancer Patients based on the “System of Health Accounts 2011”: insights into influencing factors. J Cancer.

[9] Gauttam P, Patel N, Singh B, Kaur J, Chattu V, Jakovljevic M (2021). Public Health Policy of India and COVID-19: diagnosis and prognosis of the combating response. Sustainability.

[10] Jakovljevic M, Liu Y, Cerda A, Simonyan M, Correia T, Mariita R (2021). The Global South political economy of health financing and spending landscape – history and presence. J Med Econ.

[11] Canby S, Kirca M (2022). Health expenditures (total, public and private) and per capita income in the BRICS + T: panel bootstrap causality analysis. J Econ Finance Adm Sci.

[12] Jakovljevic M, Lamnisos D, Westerman R, Chattu V, Cerda A (2022). Future health spending forecast in leading emerging BRICS markets in 2030: health policy implications. Health Policy Res Syst.

[13] Chapin A, Dieleman JL, Tsakalos G, Chang AY, Cowling K, Micah AE (2019). Past, present, and future of global health financing: a review of development assistance, government, out-of-pocket, and other private spending on health for 195 countries, 1995–2050. Lancet.

[14] Xu J, Mills A (2019). 10 years of China’s comprehensive health reform: a systems perspective. Health Policy Plan.

[15] Jin H, Jakovljevic M (2023). Fiscal decentralization and the Human Development Index: a cross-border empirical study. Sustainability.

[16] Sridhar D, Gómez E (2011). Health Financing in Brazil, Russia and India: what role does the International Community play?. Health Policy Plan.

[17] Rahman T (2008). Determinants of public health expenditure: some evidence from indian states. Appl Econ Lett.

[18] Kosaka M, Ozaki A, Kaneda Y, Saito H, Yamashita E, Murayama A, Jakovljevic M (2023). Generic drug crisis in Japan and changes leading to the collapse of universal health insurance established in 1961: the case of Kobayashi Kako Co. Ltd Cost Effectiveness and Resource Allocation.

[19] Panda B, Rout HS, Access (2020). Utilisation and Challenges of Biju Krushak Kalyan Yojana (BKKY): a Case Study from Odisha, India. J Rural Dev.

[20] Arora GK, Gumber A, Globalization (2005). Healthcare Financing in India: some emerging issues. Public Financ Manag.

[21] Hooda SK (2016). Determinants of Public Expenditure on Health in India: a Panel Data Analysis at Sub-National Level. J Quant Econ.

[22] Mulcahy P, Mahal A, McPake B, Kane S, Ghosh P, Lee J (2021). Is there an association between public spending on health and choice of healthcare providers across socioeconomic groups in India? - evidence from a national sample. Soc Sci Med.

[23] Romaniuk P, Poznanska A, Brukało K, Holecki T (2020). Health System Outcomes in BRICS Countries and their Association with the Economic Context. Front Public Health.

[24] Araujo E, Medici A, Lobo M (2021). Efficiency and sustainability of public health spending in Brazil. J Bras Econ Saúde.

[25] MV M, Sastry N, Moonesar I, Rao A (2022). Predicting Universal Healthcare through Health Financial Management for Sustainable Development in BRICS, GCC, and AUKUS Economic Blocks. Front Public Health.

[26] Jakovljevic M, Kozlova O, Makarova M, Neklyudova N, Pyshmintseva O (2023). Partial contribution of socioeconomic factors to the mortality rate of the working-age population in Russia. Healthcare..

[27] OECD. Projections of health expenditure. Health at a glance 2019: OECD Indicators. OECD Publishing. Paris; 2019. 10.1787/3d1e710c-en.

[28] Tediosi F, Finch A, Procacci C, Marten R, Missoni E (2016). BRICS countries and the global movement for universal health coverage. Health Policy Plan.

[29] OECD (2021). Reviews of Health Systems: Brazil.

[30] Rathi A, Avasthi S, Pradhan R, BRICS. & Covid-19 Casualties: Lessons from Four Continents. Centre for Public Policy Research. 2021. https://www.cppr.in/archives/brics-covid-19-casualties-lessons-from-four-continents. Accessed 30 May 2022.

[31] Kumagai N, Nishimura S, Jakovljević M (2023). Could high continuity of care (COC) have a negative impact on subjective health of hypertensive patients? A japanese perspective. Cost Eff Resource Allocation.

[32] Zhu J, Yan W, Zhu L, Liu J (2021). COVID-19 pandemic in BRICS countries and its association with socio-economic and demographic characteristics, health vulnerability, resources, and policy response. Infect Dis Poverty.

[33] Ndaguba E, Hlotywa A (2021). Public health expenditure and economic development: the case of South Africa between 1996 and 2016. Cogent Econ Finance.

[34] Dieleman JL, Campbell M, Chapin A, Eldrenkamp E, Fan VY, Haakenstad A (2017). Future and potential spending on health 2015–40: development assistance for health, and government, prepaid private, and out-of-pocket health spending in 184 countries. Lancet.

[35] Mueller M, Morgan D (2017). New insights into health financing: first results of the international data collection under the system of Health Accounts 2011 framework. Health Policy.

[36] OECD Health Statistics. OECD iLibrary. 2022. 10.1787/health-data-en. Accessed 24 June 2022.

[37] Kolassa S (2011). Combining exponential smoothing forecasts using Akaike weights. Int J Forecast.

[38] OECD/Eurostat/WHO (2017). A system of Health Accounts: revised Edition.

[39] Hyndman R, Koehler AB, Snyder R, Grose SD (2008). Forecasting with Exponential Smoothing:. The State Space Approach.

[40] Hyndman R, Koehler AB, Snyder R, Grose SD (2008). Forecast Exponential Smoothing: State Space Approach.

[41] Rao KD, Petrosyan V, Araujo EC, McIntyre D (2014). Progress towards universal health coverage in BRICS: translating economic growth into better health. Bull World Health Organ.

[42] Global Health Observatory Data Repository. World Health Organization, Geneva. 2014. http://apps.who.int/gho/data/node.main?lang=en. Accessed 25 Jan 2023.

[43] Yoon K, Kim HK, Choi M, Lee M, Jakovljevic M (2023). Analyzing the effectiveness of Data-Linked Projects for Health Promotion in Public Health Centers of South Korea. Risk Manag Healthc Policy.

[44] Berman PA (1998). Rethinking health care systems: private health care provision in India. World Dev.

[45] Ivins C (2013). Inequality matters: BRICS inequalities fact sheet. Oxfam Policy and Practice: Climate Change and Resilience.

[46] Su CW, Huang SW, Tao R, Haris M (2021). Does economic overheating provide positive feedback on Population Health? Evidence from BRICS and ASEAN Countries. Front Public Health.

[47] Mason A, Lee R, Abrigo M (2017). Support ratios and demographic dividends: estimates for the world.

[48] Sahoo PM, Rout HS, Jakovljevic M (2023). Contemporary Universal Health Coverage in India – The Case of Federal State of Odisha (Orissa). Risk Manag Healthc Policy.

[49] World Population Review. 2023 World Population by Country. US Census Beureau. 2023. https://worldpopulationreview.com/. Accessed 25 Jan 2023.

[50] Watt NF, Gomez EJ, McKee M (2014). Global health in foreign policy–and foreign policy in health? Evidence from the BRICS. Health Policy Plan.

[51] Sahoo PM, Rout HS, Jakovljevic M (2023). Consequences of India’s population aging to its healthcare financing and provision. J Med Econ.

[52] Demir S, Demir H, Karaduman C, Cetin M (2023). Environmental quality and health expenditures efciency in Türkiye: the role of natural resources. Environ Sci Pollut Res.

